# “Psychosomatic consultation in the workplace” – a new model of care at the interface of company-supported mental health care and consultation-liaison psychosomatics: design of a mixed methods implementation study

**DOI:** 10.1186/1471-2458-12-780

**Published:** 2012-09-13

**Authors:** Eva Rothermund, Reinhold Kilian, Michael Hoelzer, Dorothea Mayer, Daniel Mauss, Marc Krueger, Monika A Rieger, Harald Guendel

**Affiliations:** 1 Department of Psychosomatic Medicine, University Clinic of Ulm, Ulm University, Am Hochstraess 8, 89081, Ulm, Germany; 2 Manager AG, Versorgungsforschung, University of Ulm, BKH GuLudwig-Heilmeyer-Straße 2, 89312, Guenzburg, Germany; 3 Division of Psychosomatic Medicine of the ZfP Suedwuerttemberg, Sonnenbergklinik, Christian-Belser-Straße 79, 70597, Stuttgart, Germany; 4 Daimler AG Werksaerztlicher Dienst Health and Safety Sindelfingen, 71059, Sindelfingen, Germany; 5 Occupational Health Department, Allianz AG, Koeniginstr. 28, 80802, Munich, Germany; 6 CASSIDIAN, Betriebsmedizinischer Dienst, Landshuterstr. 26, 85716, Unterschleissheim, Germany; 7 Institute for Occupational and Social Medicine, and Health Services Research, Universitaetsklinikum Tuebingen, Wilhelmstraße 27, 72074, Tuebingen, Germany

**Keywords:** Company supported mental health care, Depression, Anxiety, Somatisation, Burnout syndrome, Qualitative design, Explorative study

## Abstract

**Background:**

Mental health issues are gaining in importance in society and the economic system. At the same time, the accessibility and stigmatisation of the mental health care system in Germany can obstruct help-seeking behavior and delay early psychotherapeutic interventions. Therefore, new models of care are being established at the interface of company-supported health promotion and conventional health insurance sponsored outpatient care for people developing mental illnesses. Two large industrial companies, in cooperation with two psychosomatic clinics, have recently established a model of “psychosomatic consultation in the workplace“. This new model of care offers the opportunity for a first psychotherapeutic door to door consultation with occupational medicine within the industrial workplace. The main empirical goals of this study are:

1) Describing the differences between patients who use this new diagnostic and therapeutic offer within the industrial workplace vs. patients who visit a conventional regional outpatient clinic, especially in regard to symptom duration and severity, work ability, and demographic characteristics, and

2) A first evaluation of how patients may benefit more from this new model of care compared to those first seen by standard outpatient care.

In the qualitative part of the study, occupational physicians, psychosomatic therapists, involved personnel and select employees of the involved companies will be asked to comment on their experiences with this new approach.

**Methods/Design:**

The implementation study will take place in Ulm and in Stuttgart, with each site looking at one regional conventional psychosomatic outpatient clinic and one psychosomatic consultation offer within the workplace. 70 consecutive patients in each setting will be recruited (overall n = 280). For the cross-sectional study and pre-post comparison we will use established and validated survey instruments (PHQ, SF-12, WAI, MBI, IS) as well as standardized questions about health care use. For data analysis, we will use uni- and multivariate analytical methods. Qualitative data analysis (expert interviews) will be carried out using Mayring’s content analysis method.

**Discussion:**

The results of this study have the potential to provide evidence-based knowledge about an innovative model of psychotherapeutic outpatient care and to further promote tailored solutions for early psychotherapeutic interventions within the worksite.

**Trial Registration:**

DRKS00003184

## Background

In Germany, absenteeism due to mental health issues is on the rise [1]. The proportion of mental health diagnoses related to early retirement is currently 40%, constituting the largest diagnostic group [2]. The reasons for the rise in this category of diagnoses are diverse and often involve a potent combination of personal and occupational stress [3]. Demands for mobility and flexibility in an already intense workplace are increasing when having and keeping a job is often uncertain [4,5]. Although the concept of mental or psychosomatic disorders has become more acceptable in society, the affected usually still have to overcome a "fear threshold" due to stigmatization [6]. Conventional access to the mental health care system, especially for outpatient psychotherapy, is usually connected to long waiting periods which primarily could raise this “fear threshold” and ultimately increase the likelihood of illness chronification [7].

Due to the increasing prevalence of rates of mental disorders among employees and the subsequent shortage of qualified staff [8], most companies in Germany offer workplace health promotion programs and stress management interventions for their employees. These interventionsare intended to reinforce preventive and salutogenic behaviors, while some also address unfavorable working conditions. Although these interventions seem to be effective, they are primarily geared to healthy employees, and therefore fail to adequately address the needs of those who already suffer from mild but clinically relevant psychological disturbances or fully developed mental illnesses [9]. Thus, occupational health care professionals, human resources staff and affected employees need easily accessible and specifically tailored psychotherapeutic consultations for work-related mental health issues. These groups increasingly enforce the creation and implementation of new models of mental health care in the workplace. The specific model considered in detail here is “Psychosomatic Consultation in the Workplace”, a kind of specific consultation-liaison psychosomatic. In Germany, the field of psychosomatic medicine and psychotherapy is a separate medical speciality next to psychiatry, focussing on the relationships of psychological, social and behavioral factors on bodily processes as well as on mental health. Psychodynamic as well as cognitive-behavioral psychotherapy constitutes the main treatment procedure.

Currently, little is known about the profile of utilizers, or about the effects of this new model of care. Therefore, our study aims to assess the following research questions:

1. Are there differences in demographic, clinical and psychometric characteristics between patients who use the consultation offer in the workplace versus patients who use conventional outpatient care? Is this new model of mental health care successful in reaching employees suffering from psychological disorders earlier in the course of their illness, compared to patients who seek help in conventional outpatient care?

2. What changes occur within a three-month follow-up period after using the offer "psychosomatic consultation at the workplace"? Are they different from those of a conventional outpatient clinic?

3. What is the feedback (i.e. appraisals, attitudes, criticism) of persons who are involved with the offer "psychosomatic consultation in the workplace"?

## Methods/Design

### Intervention

The investigation will take place in two areas in southern Germany, each with an established psychosomatic outpatient clinic and a company providing the newly established model of a "psychosomatic consultation in the workplace". This translates into two regions with two groups each, making a total of four settings (2x2 design). 12 weeks after the first contact (baseline, T1) a postal follow-up survey will take place (follow-up, T2). Participants will be consecutively recruited from November 2011 to September 2012.

The offer of a “psychosomatic consultation in the workplace” is based on a consultation-liason model developed in the general hospital and specifically tailored to the needs of occupational medicine within the workplace [10].

This new model of mental health care has been established on a step by step basis within a large automobile manufacturer in cooperation with a local psychosomatic clinic over the last six years [11]. The psychosomatic consultation offer at a company for security systems started in January 2011, in cooperation with the Department of Psychosomatic Medicine and Psychotherapy, University of Ulm. The employees in the first workplace setting (Stuttgart) are mainly referred to psychosomatic medicine by the occupational physicians or the social worker. The employees at the second setting (Ulm) can directly participate in this offer. For all groups (outpatient and workplace), the offer includes the initial consultation, diagnosis, indication, crisis intervention (if needed) as well as support for referral into the existing secondary treatment system.

The Medical Ethics Board of the University of Ulm has given approval for the study. Patient participation is voluntary. Patients can leave the study at any time without consequences. All patients sign an informed consent form. If a participant drops out, care will be continued.

### Study design

**Figure 1:**
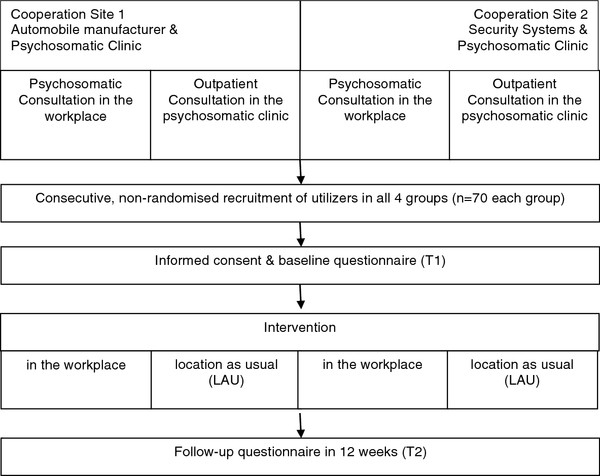
**Study design. **Overview of the quantitative part of the study design.

A mixed methods design [12,13] will be utilized to explore the new care model of "psychosomatic consultation in the workplace" as compared to conventional outpatient clinics. The mixed methods design incorporates a cross-sectional study (research question 1), a second quantitative portion with pre-post comparison (research question 2) (see Figure 1) and a qualitative section (research question 3).

### Sample size and power calculations

Statistical power analyses was conducted with regard to the work ability index (WAI) [14] as primary outcome. In our regression model we assume that the condition „intervention in the workplace/in the psychosomatic clinic“explains a variance of 5%. This means that at a statistical significance of 5% and a power of 95%, we will need 110 participants in each group (workplace and clinic) with a total sample size of n = 220. Bearing in mind a drop out rate of 20% we will include 280 patients.

The quantities for the main question (research question 1) in this explorative study result from the foreseen time schedule during the funding period and the feasibility of recruitment. We expect a high rate of participation since the "psychosomatic consultation in the workplace" so far has been greatly accepted and appreciated by the employees. In addition, the employees are assured that the collected data will be kept confidential under the supervision of the research group at the university. To increase the response rate for the follow-up survey, there will be a reminder mailing or a phone call.

There are 14 variables to be measured. The two intervention groups consist of employees who use this service within the 10 month recruitment period. One company has a total of 70,000 employees at the participating sites. The other company has about 3,000 employees. The ratio of male to female employees in both companies is about 4:1 with an average age of 43 years. The employees are unevenly distributed to work in research, development, production and administration.

### Instruments and selected items used in the quantitative analysis

The questionnaires at T1 and T2 are both structured into five sections.

At both time points, we will examine the nature and severity of mental illness through self administered instruments resulting in discrete sumscores or subscale sumscores: the Patient Health Questionnaire (PHQ) [15], health-related quality of life (Short-Form 12, SF-12) [16], ability to work (work ability index, WAI) [14], burnout syndrome based on work-related stress (Maslach Burnout Inventory, MBI) [17], cognitive and emotional irritation (irritation scale, IS) [18] and working conditions (using the short questionnaire for work analysis, KFZA [19]).

At T1 we ask for demographic variables (age, gender, marital status, number of own children and the number of children in a household, educational and occupational status) as well as the number of previous contacts with the mental health care/psychotherapeutic system. The nature and frequency of previous psychotherapeutic treatments will be specified in months and standardized. The duration of the chief complaint will be recorded by the interviewer and also expressed in months.

The questionnaire of T2 has additional questions about the follow-up of the patient in the psychotherapeutic system, the implementation of the follow-up recommendations and patient satisfaction (ZUF-8) [20] (Table 1).

**Table 1:** Target assessments and instruments

		**Time of implementation**	
**Target assessment**	**Instrument**	**T1**	**T2**
Demographic data	Individual items	x	
Previous contacts with the psychotherapeutic system	Individual items	x	
Work ability	WAI	x	x
Psychosomatic diagnosis, nature and severity of the illness	PHQ	x	x
Length of symptoms	Individual items	x	
Health-related quality of life	SF-12	x	x
Burnout syndrome based on work-related stress	MBI	x	x
Cognitive and emotional irritation based on work-related stress	IS	x	x
Working conditions	KFZA	x	x
Follow-ups in the psychotherapeutic system	Individual items		x
Patient satisfaction	ZUF-8		x

Overview of target assessments and corresponding psychometric instruments. For each instrument the time of implementation is indicated. T1 stands for baseline/before the intervention, T2 is defined as 12 weeks after T1.

### Statistical analysis

Research question 1 : *Are there differences in demographic, clinical and psychometric characteristics between patients who use the consultation offer in the workplace* versus *patients who use the established outpatient care?*

The differences between treatment groups will be examined by means of logistic regression analysis (in the form of binomial and multinomial models) including age, sex, educational level, occupational category, using the number of previous contacts with psychiatric-psychosomatic services as independent variables. The results of the logistic regression models will be used to estimate the propensity scores for the conditional probability of being referred to a particular treatment group.

The factors of interest of research question 1 are the type and severity of the disease (PHQ) the duration of symptoms, the health-related quality of life (SF-12), work ability (WAI), work-related stress effects (MBI, IS), work requirements (KFZA), the number of previous contacts with the mental health care system as well as demographic factors (Table 1). The demographics may be incorporated to the model independently or in terms of propensity scores.

By using multivariate analysis (latent class analysis = LCA) techniques it is possible to discern which patients with specific combinations of traits fit into certain clusters and determine the effect the particular setting has on the patients’ assignment in each particular cluster. The multivariate analysis is performed using the General Mixture Model (GMM) approach [21,22]. The basis here is the relationship between various characteristics of a limited number of categorical latent classes. The number of latent classes can be determined via various statistical methods (Bayesian Information Criterion, Akaike Information Criterion, Lo-Mendel-Rubin Test). By extending this model to a conditional latent class model, the effects of covariates on the likelihood of class membership can be estimated. The covariates in this study are the respective treatment settings, demographic data and job-specific characteristics of the responders.

Research question 2: *What are the changes 12 weeks after initially using the offer "psychosomatic consultation in the workplace" and how are they different from those in the control group?*

At time point T2, we will collect as primary outcome work ability (WAI). As secondary outcome we will collect the following target characteristics: type and severity of the disease (PHQ), health-related quality of life (SF-12), work-related stress effects (MBI, IS) and work requirements (KFZA). The main point of research question 2 is to detect whether or not there are significant differences between T1 and T2. The only newly collected data for T2 is patient satisfaction which will be analyzed descriptively. The effects of the setting on the change of the clinical characteristics will be analyzed by multivariate linear regression models with membership in the setting as independent variable and controlling for selection bias by including the propensity scores for the different treatment groups.

#### Qualitative analysis

The qualitative analysis will be performed by conducting expert interviews with representatives from three participatory groups (occupational health professionals, work councils and human resources professionals) within the companies. To qualify as an expert, the person would have to be involved in the implementation of the "psychosomatic consultation in the workplace." For both companies, this will yield about 3-5 experts per group and company.

The target characteristics and assessment method for research question 3: *What feedback is provided for persons involved with the offer for "psychosomatic consultation in the workplace" for the new model of care?*

We will use expert interviews to explore how the new offer is perceived. Expert interviews as a distinct form of semi-structured interviews are described by Meuser and Nagel (2002) [23,24]. Unlike the biographical interviews, the questions are not geared towards the personal traits of the interviewees but at their expert knowledge in their respective fields. The focus on the experts' status significantly and effectively limits the range of potentially relevant information that the respondent can provide [24].

The interviews are intended to encourage the experts to critically reflect on the implemented offer - the psychosomatic consultation in the workplace - and to report on their experiences, impressions and attitudes concerning changes in their respective company-supported health care systems. According to P. Mayring the texts will be transcribed and analyzed using qualitative content analysis methods. This technique will enable us to make inferences about the attitudes and impressions of the interviewees [25]. Text management will be supported by atlas.ti.

### Instruments

#### The work ability index (WAI) (short version)

The Work Ability Index (WAI) [14] is a quantitative instrument used to assess current and future work ability as well as work demand management based on behavioral measures. In our study, we are using the short version with 7 items. It has high reliability and validity.

#### Patient health questionnaire (PHQ), German version

The PHQ is an ideal and reliable tool to assess the most common mental disorders somatisation (15 items), anxiety (7 items) and depression (9 items) via self-assessment. The responses measure the frequency or the presence of symptoms with 2, 3 or 4 levels of severity. The interpretation of the PHQ is based on the diagnostic criteria of the DSM-IV and ICD-10, using a summative score between 0-30 with a 4-level severity scale of minimal, mild, moderate or severe. Patients who reach a cut off point of 10 or more are likely to require treatment. The PHQ is assumed to be objective because it is standardized in terms of its implementation and evaluation. The level of internal consistency for the continuous scale of the depression module is r = 0.88 and for the somatisation module it is r = 0.79. The test-retest reliability of the depression module lies between ICC = 0.81 and ICC = 0.96 (10). The criterion validity of the German version of the PHQ was identified as the “gold-standard” after being tested on 528 patients with reference to the "Structured Clinical Interview for DSM-IV”. The depression scale of the PHQ is also sensitive to change, making it a useful tool in longitudinal studies [15].

#### Health-related quality of life: SF-12

The SF-12 is the validated short version of the SF-36, an instrument that measures the health-related quality of life of patients. The 12 individual items record 8 dimensions, which can be conceptually sorted in terms of "physical health" and "mental health." The calculated scores can reach values of between 0 and 100 points, with low values reflecting poor health and higher values indicating better health [16].

#### Maslach Burnout Inventory (MBI), German general version (MBI-GS-D)

The MBI is used to assess burnout syndrome as a manifestation of mental exhaustion [17]. Although it overlaps with the depressive syndrome (ICD-10), it demonstrates a useful model of cause and effect. The phenomenon of burnout was first described in the helping professions. In the present study, we use the general version, which has been validated with 16 items (MBI-GS-D). Thus, the method allows a diagnosis of the three components of burnout: emotional exhaustion (EE), depersonalization (DP) and (reduced) personal accomplishment (PA). The items are classified according to frequency. These were tested and validated in several business sectors and occupations.

#### The irritation scale (IS)

Irritation is defined as a subjectively perceived emotional and cognitive strain in occupational contexts. The Irritation Scale, in particular, is recommended for application in occupational contexts. It can be used for evaluating interventions, research on stress at work, and individual counselling. In addition, irritation has been shown to be a precursor to other impairments. The irritation scale is a subjective tool with proven reliability and validity [18].

#### KFZA: analyzing work requirements (KFZA)

The KFZA is a method of determining working conditions. We used the short form based on the selection of 26 items from the long version. This is a time-efficient and subjective screening instrument to detect positive and negative influences of industrial and organizational structure. The results include aspects of work content, resources, stressors and the organizational climate and yield information about health promotion programs [19].

#### Patient satisfaction (ZUF-8)

The ZUF-8 (created by J. Schmidt, F. Lamprecht and W. Wittmann) is a method for globally detecting patient satisfaction with confirmed reliability and validity. It consists of eight items that have four answer choices without a "neutral" position. The individual items also have face validity. The answers each have a value of 1 to 4. Four of the eight items (items 1, 3, 6 and 7) are pooled negatively. After reversing the negatively poled items, the scores of all eight items are added (summative scores range from 8 to 32). High summative scores indicate high patient satisfaction, and vice versa [20].

### Inclusion criteria

The only inclusion criterion is that the respondents must be at least 18 years of age. Other inclusion criteria are not relevant since we are recruiting consecutively and this is an explorative study.

## Discussion

Our study set out to investigate the utilization of a new and interdisciplinary model of care at the interface of occupational and psychosomatic medicine. The main objective is to identify different types of patients with specific combinations of traits with regard to the setting (outpatient clinics vs. workplace). A pre-post test 12 weeks after consultation will detect changes in ability to work, disease severity and quality of life compared with the first consultation. We will describe the extent to which psychotherapeutic recommendations have been followed by the patients. Furthermore, we evaluate patient satisfaction. Using the instrument of qualitative interviews we want to work out the appraisals, attitudes, and experiences of employees, occupational physicians, therapists and other company personnel with this new model of care.

### Strengths and weaknesses

The main strength of our study is that, except for one non-empirical article [11], there is little data in this field. We will evaluate whether or not we will reach different types of patients if we, as health professionals, initially see patients at their workplace and not within the traditional setting of a psychosomatic outpatient clinic. The best way to investigate this question is an explorative design with consecutive recruitment of patients. Therefore, we do not match groups for age, gender or desease severity.

A disadvantage of the explorative design is the inability to compute effect sizes. To be able to obtain utilization data of our model under routine conditions, we decided to choose a non-randomized controlled design. Another limitation of the study is the recruitment of a sample size of 280 participants in the allotted time of 10 months. Most studies in this field have problems recruiting participants. In contrast to the other studies, however, we expect a high rate of participation because the "psychosomatic consultation in the workplace" has been appreciated and well-received by the employees so far. In addition, the employees are assured that the collected data will be kept confidential under the supervision of the research group at the university. Another critical issue might be a high dropout rate at follow-up which we will attempt to minimize with a reminder phone call, email or letter. A long-term follow-up survey (i.e. at 6 months after T1) would be reasonable, but cannot be realized within the funding period. We plan to investigate this issue in a future project.

### Relevance of the study

A lack of easy accessability, as well as stigmatisation of the mental health care system [26], obstructs help-seeking behavior. In Germany, 50% - 90% of individuals with psychosomatic disorders do not manage to obtain professional care, as a recent report on behalf of the National Association of Statutory Health Insurance Physicians (KBV) revealed [27,28]. These aspects, among others, may contribute to the chronification of common mental disorders such as depression, anxiety and adjustment disorder, as well as functional somatic disorders. Therefore, common mental disorders should be treated within the early stages of onset, as they are known to be especially responsive to treatment during the early phases of illness [29]. In addition, a recent study showed that even if the employees’ home life is stressful, tackling workplace stress is likely to improve employees’ psychological health [30]. These facts validate the need for brief, easily accessible and tailored interventions at the workplace.

With the model “psychosomatic consultation in the workplace,” we aim to offer an integrative, timely and easily accessible first consultation to affected employees. The stigma of mental disorder is intended to be minimized by bringing the psychosomatic consultant to the patient instead of vice versa. Furthermore, the psychosomatic consultation is embedded within occupational health facilities at the workplace. These aspects are likely to make the consultation at the workplace an instrument to reduce chronification.

In this study, we will describe the utilizers of a new model of mental health care in the workplace. This model follows numerous other concepts of work-related programs, such as return to work interventions [31,32], stress prevention training [9,33,34] and interventions for healthy management [35,36]. “Psychosomatic consultation in the workplace” addresses those workers and employees who still have the ability to go to work. It focuses on improving work ability of affected individuals, and hence, can be regarded as a form of secondary and tertiary prevention.

## Abbreviations

DP: Depersonalization; DSM IV: Diagnostic and Statistical Manual of Mental Disorders 4th edition; EE: Emotional Exhaustion; ICC: Intraclass Correlation; ICD-10: International Classification of Diseases and Related Health Problems, 10th edition; IS: Irritation Scale; KBV: National Association of Statutory Health Insurance Physicians; KFZA: Short Questionnaire for Work Analysis; LAU: Location as Usual; MBI: Maslach Burnout Inventory; PA: personal accomplishment; PHQ: Patient Health Questionnaire; SF-12: Short Form 12; T1: Timepoint 1, baseline; T2: Timepoint 2, follow up; WAI: Work Ability Index; ZUF-8: Patient Satisfaction Scale, 8 Items.

## Competing interests

For the qualitative analysis, the interviews will be conducted by a third person involved in the study since the principle investigator is also a participating physician in the study and would confound the results. Beyond that, the authors declare no other competing interests.

## Authors’ contributions

ER, RK, MH and HG designed the study and drafted it. MAR was involved in drafting the manuscript and revising it critically. DM1, DM2 and MK adapted and implemented the protocol for their companies and coordinated local data collection. All authors read and approved the final manuscript.

## Pre-publication history

The pre-publication history for this paper can be accessed here:

http://www.biomedcentral.com/1471-2458/12/780/prepub
